# Prolonged Postdiapause: Influence on some Indicators of Carbohydrate and Lipid Metabolism of the Red Mason Bee, *Osmia rufa*

**DOI:** 10.1673/031.013.7701

**Published:** 2013-08-10

**Authors:** Kamila Dmochowska, Karol Giejdasz, Monika Fliszkiewicz, Krystyna Żółtowska

**Affiliations:** 1 Department of Biochemistry, Faculty of Biology, University of Warmia and Mazury, Olsztyn, Poland; 2 Department of Apidology, Institute of Zoology, Poznań University of Life Sciences, Poland

**Keywords:** diapause, enzymes, insect metabolism, solitary bees

## Abstract

Bees of the genus *Osmia* are being used in crop pollination at an increasing rate. However, a short life expectancy of adult individuals limits the feasibility of their use. Cocoons of the red mason bee, *Osmia rufa* L. (Hymenoptera: Megachilidae), can be stored at 4° C in a postdiapause state, and adult bees can be used for pollination outside their natural flight period. The period of storage in this form has an unfavorable influence on the survival rate, life expectancy, and fertility of the bee. It was suggested that the negative results are connected with exhaustion of energy reserves. To test this hypothesis, the present study examined the contents of protein, carbohydrates, lipids, and the activities of some enzymes, and their degradation in red mason bees that emerged in spring according to their biological clock and in summer after elongated diapause. It was found that postdiapause artificially elongated by 3 months caused significant decreases in body weight, total sugar, glycogen, lipids, and protein content in *O. rufa.* Glucose level was highest in bees that emerged in the summer, which was coincident with increased activities of maltase and trehalase. The activities of sucrase and cellobiase were not changed, while amylase activity was considerably decreased. The activities of triacylglycerols lipase and C2, C4, C10 carboxylesterases were highest in bees that emerged in July. Low temperatures restrict *O. rufa* emergence, and during prolonged postdiapause, metabolic processes lead to significant reductions of structural and energetic compounds.

## Introduction

Diapause is a fundamental process that allows insects to synchronize their life cycle with seasonal weather changes. Obligatory diapause occurs in the red mason bee, *Osmia rufa* L. (Hymenoptera: Megachilidae), which allows them to survive the winters. This solitary bee overwinters as a fully enclosed, cocooned, and unfed imago. Its diapause is not dependent on photoperiod. The duration of the overwintering period depends on the temperature and is important in both bee survivability and bee usability in crop pollination in the following vegetation year (Bosch and Blas 1994; Bosch and Kemp 2004). The metabolic rate of *O. rufa* decreases in late summer, just after transforming into an imago. This decreased metabolism rate is a very important phenomenon, as survival of the winter period depends on the amount of food stored in the organism during the larval stage (Bosh et al. 2010).

According to the general diapause model (Kostal 2006), *O. rufa* overwintering consists of 2 phases, diapause and postdiapause quiescence. This concept of diapause in *Osmia* genus has been confirmed by many studies (Bosh and Kemp 2003; Kemp et al. 2004; Krunic and Stanisavljevic 2006; Sgolastra et al. 2010). During diapause, supercooling point values in *O. rufa* decline (Krunic and Stanisavljevic 2006). Placing diapausing *O. rufa* at 20° C leads to their death. Diapause last about 100 days and seems to be independent of the temperature variation. After this period, bees of *Osmia* genus can develop normally, but their development is inhibited by the temperature (Sgolastra et al. 2010). This period is called the stage of postdiapause quiescence. In the beginning of postdiapause in *Osmia cornuta* and *O. rufa,* their supercooling point value begins to grow until spring (Krunic and Stanisavljevic 2006). This growth could be the result of a decrease in protective compounds, such as glycerol, sorbitol, trehalose, etc. (Storey and Storey 1991).

It is known that *O. rufa* in can be kept in postdiapause quiescence for a long time by being stored in a cooler. This practice allows beekeepers to activate bees and use them for pollination at the desired time. For example, the stored bees can be used to pollinate plants that flower in the summer, a time when under natural conditions the bees would have already finished their flight period.

Artificially prolonging postdiapause in *O. rufa* has an unfavorable effect on their survivability and fertility (Bosch and Blas 1994; Giejdasz and Wilkaniec 1998; Bosh and Kemp 2003, 2004; Sgolastra et al. 2010). Futhermore, prolonging wintering can cause a partial loss in the effectiveness of antioxidant systems (Dmochowska et al. 2012). It seems that the main cause of these undesired occurrences is the exhaustion of reserve substances. This suggestion is supported by the fact that bees of *Osmia* genus with larger body weights have a higher overwintering survival rate than lighter bees (Tepedino and Torchio 1982; Bosh and Kemp 2004); however, this hypothesis had not been examined experimentally on the molecular level until the present study.

In insects, the predominant materials stored in the fat body are lipids, mainly as triacylglycerols and polysaccharide-glycogen (Canavoso et al. 2001; Arrese and Soulages 2010). Besides the energetic value lipids and carbohydrates play in insects wintering at below-zero temperatures, they also play an important role as the substrates for synthesis of cryoprotectants as glycerol, trehalose, or other polyols (Hahn and Delinger 2007).

The aim of the present study was to determine and compare selected biochemical parameters of newly-emerged *O. rufa* after a natural overwintering period (in April) and an artificially prolonged postdiapause quiescence under laboratory conditions (in July). Body weight, total protein, carbohydrates and lipid contents, glycogen and glucose levels, and the activity of chosen enzymes of lipid and carbohydrate metabolism were analyzed. The results obtained will help determine how elongation of postdiapause quiescence influences the energetic stores of *O. rufa* and how the metabolism of *O. rufa* is optimized for its role as a pollinator. It should be highlighted that this is the first report on any elements of lipid and sugar metabolism in *O. rufa.*

## Materials and Methods

### Bees

*O. rufa* were reared in artificial reed tube nests. The *O. rufa* cocoons and the artificial nests were placed in nesting shelters situated at the Swadzim Biological Station of the Department of Apidology, Poznań University of Life Sciences, Poznań, Poland. During the nesting period (from April to June 2009), *O. rufa* females occupied the nest tubes, which were transferred to the laboratory in February. In the laboratory, the nest tubes were dismantled, and the adult bees in cocoons were removed from nest cells. The wintering bees were kept in a SANYO cooler (www.us.sanyo.com) at 4° C. On April 5^th^ and July 2^nd^, randomly selected cocoons were placed in an incubator at 25° C for emergence.

### Sample preparation

The emerged bees were weighed, then placed in eppendorf tubes and immediately frozen in liquid nitrogen. Until analyses, the material was stored at -71° C. Forty females were randomly selected from bees that emerged in April or July. They were divided into 20 samples, each of 2 individuals). The samples were homogenized in an ice bath for two minutes with 0.9% NaCl at 1:10 (w/v) ratio. The homogenate was centrifuged at 4° C for 15 minutes at 15000× *g.* The supernatant was carefully collected from under the fatty layer for analysis of proteins, total sugars, glucose and glycogen content, and the activity of αamylase, maltase, sucrase, trehalase, cellobiase, triacylglicerol lipase, and carboxylesterases. Lipids were extracted separately with a mixture of chloroform and methanol (2:1) according to Folch et al. (1957).

### Biochemical assay

The protein content was assayed spectrophotometrically (A_280_) using a Nano-Drop apparatus (www.nanodrop.com) and NanoDrop 1000 version 3.6.0 software.

Total carbohydrate content was assayed using the anthrone method according to Roe (1955). To 1 mL of reagent was added 0.5 mL of extract (first diluted 20 times with deionized water). After 14 minutes of incubation at 95° C, samples were chilled, and absorbance at 620 nm was measured. Total carbohydrate content was expressed as mg/g of fresh body weight.

Glucose was assayed using the enzymatic method, using Liquick Cor-GLUCOSE 500 kit (Cormay, www.pzcormay.pl) according to the manufacturer's instructions. 10 µl of extract was added to 1 mL of 1-GLUCOSE reagent. Glucose level was expressed as µg/100 mg of fresh body weight.

Glycogen level was isolated from the extract by the micro-method described by Sölling and Esmann (1975). A 20 µl sample was pipetted on square Whatman No. 3 filter paper (10 mm side). In the next step, glycogen was precipitated by addition of 5 mL of 10% trichloroacetic acid in 70% ethanol, and then rinsed 3 times for 20 minutes with 5 mL of ethanol. Finally, squares were rinsed in cold acetone for 10 minutes, dried, and cut in to small pieces to fit in the NanoDrop test tube. The 0.5 mL 0.2 M acetate buffer (pH 4.8) and 30 µl amyloglucosidase (25.8 mU) (cat. nr A-7255, Sigma Aldrich, www.sigmaaldrich.com) were added to each tube. Mixtures were incubated 15 minutes at 55° C with careful shaking. At the same time, the probe of the standard solution of glycogen (5 mg/mL) was treated in an identical manner. Glucose released from glycogen by amyloglucosidase was determined by the enzymatic method. Results were expressed as µg of glucose per g of tissue.

The activity of α-amylase was assayed with a modified Caraway (1959) method. The incubation mixtures contained 50 µl extract, 0.85 mL 0.2 M acetate buffer (pH 4.8), and 0.1 mL starch solution (0.75%). The incubation lasted 120 minutes at 37° C. After this time, 4 mL iodine solution was added. For every sample, a control was prepared, which was not incubated. The activity of enzymes was expressed by mg of starch decomposed during 1 hr of incubation at 37° C per 1 mg of protein.

The activity of disaccharidases, maltase, sucrase, trehalase, and cellobiase, was assayed by Dahlqvist's (1968) method. The activities were assayed by measuring the amount of glucose released by these enzymes from their specific substrates: maltose, sucrose, trehalose, cellobiose, respectively. The assay mixture contained: 0.380 mL 0.2 M acetate buffer (pH 5.4), 20 µl of extract, and 0.1 mL 50 mM suitable substrate. The incubation lasted one hour at 37° C. The releasing glucose was determined by enzymatic method. The enzymatic activities were expressed in international enzymatic units (U).

The activity of triacylglicerol lipase was assayed by the Jurado et al. (2006) method. 100 µl extract was added to 1 mL of tributyrin emulsion. Samples were incubated for 2 hours at 37° C and titrated with 0.01 M NaOH. The activity of lipase was expressed as nmol of fatty acids released during 60 minutes of incubation at 37° C per 1 mg of protein.

The activity of carboxylesterases were assayed with the modified Walz and Schwack (2007) method. 4-Nitrophenyl-acetate (C2), 4-Nitrophenyl-butyrate (C4), or 4-Nitrophenyl-decanoate (C10) (all from Sigma Aldrich) were used as the substrate for esterases. The incubation mixtures contained 50 µl extract, 0.43 mL Teorell—Stenhagen buffer (pH 7.88), and 20 µl 1 mmole substrate. Samples were mixed, and absorbance at 405 nm was measured. After 3 minutes, the measurement was repeated. Carboxylesterases activities were expressed in international units (U) per mg of protein.

Lipid content was assayed by the sulfo-phospho-vanilin reaction (Frings et al. 1972). Lipid precipitate was dissolved with absolute ethanol (75 µl/100 mg fresh body weight). 0.2 mL concentrated sulphuric acid was added to 20 µl lipid solution. Samples were placed in boiling water for 10 minutes and then were cooled. 10 mL sulfo-phospho-vanilin reagent was added, and after 15 minutes of incubation at 37° C, the mixture was chilled, and absorbance was measured at 540 nm. Lipid content was expressed as mg per 100 mg of fresh body weight.

**Figure 1.**
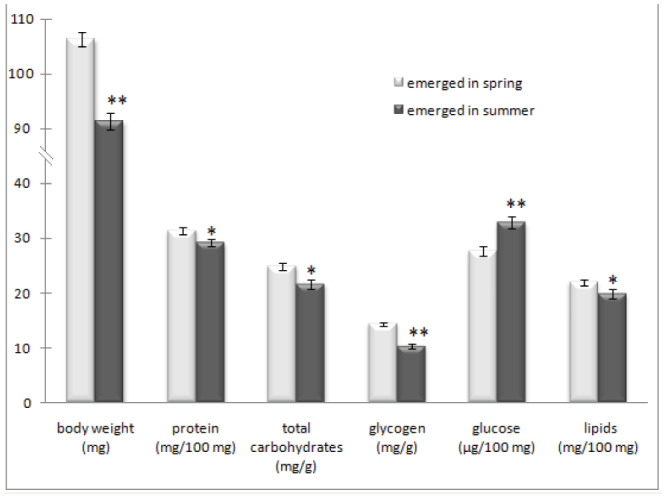
Body weight and values of selected biochemical parameters of *Osmia rufa* (mean ± SE) that emerged in spring and summer. Asterisks at the top of bars indicate significant differences between means of body weight and chemical compounds from bees emerged in spring and summer: * *p* < 0.05, ** *p* < 0.001. High quality figures are available online.

All analyses were performed in 20 samples. All samples were tested in triplicate.

### Statistical analysis

The obtained results were statistically analyzed using Statistica 9 software (StatSoft Inc., www.statsoft.pl) at the significance level *p* < 0.05. Average body weights, protein, sugar, glucose, glycogen and lipid content, and the activities of sucrase and C10 esterase were compared with Student's *t*-test. Due to non-homogeneity of variances of mean activities of amylase, cellobiase, maltase, trehalase, lipase, and C2 and C4 esterases, comparison of the mean values was performed using a *t*-test with separate variance analysis, the Cochran and Cox test.

## Results

The obtained results for weight and chemical composition of *O. rufa* bodies are shown in Figure 1. Lipids constituted about 20% of the fresh weight of the emerged females, and carbohydrates only about 2%. Glycogen constituted almost half of the all carbohydrate pool. Free glucose was in very small quantities (0.027% of body weight). The proteins constituted about 30% of the body weight (Figure 1). By comparing the results for bees emerged in April and July, it was found that body weight, protein content, total sugar content, and glycogen and lipid level were significantly higher in *O. rufa* that emerged in April than those that emerged in July. On the contrary, glucose level was higher in insects that emerged in the summer (Figure 1). A considerable decrease (around 14%) was noticed in the body weight of bees that emerged in July in comparison to those that emerged in April. The general loss of carbohydrates was greater (∼13.2%) than lipids (∼9.4%) and proteins (∼7.1%).

**Figure 2.**
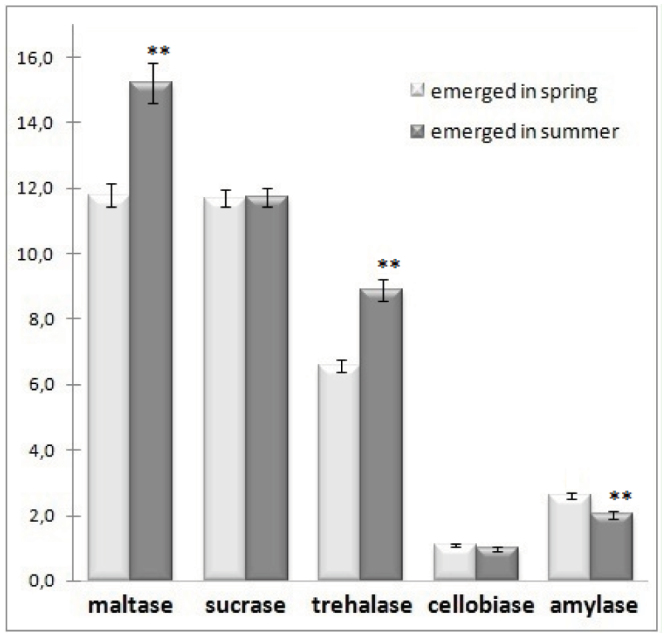
The activity of disacharidases (mU/mg) and α-amylase µg starch/mg) in *Osmia rufa* (mean ± SE). Asterisks at the top of bars indicate significant differences between means of activity enzyme from bees emerged in spring and summer: ** *p* < 0.001. High quality figures are available online.

The activity of α-amylase in newly emerged bees from both time periods was not high. However, a higher α-amylase activity was observed in *O. rufa* that emerged in spring compared to those that emerged in the summer. Among the studied disaccharidases, the highest activity was observed for maltase, followed by sucrase and trehalase. Cellobiase showed the lowest activity among the studied disaccharidases (Figure 2). The activities of maltase and trehalase were significantly higher in bees emerged in July. The activities of sucrase and cellobiase were similar in bees from both times of emergence (Figure 2).

**Figure 3.**
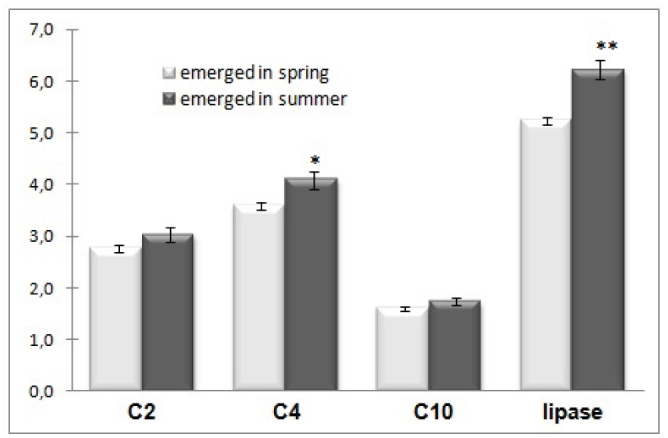
The activity of carboxylesterases C2, C4, C10 (U/mg), and lipase (nmol/mg) in *Osmia rufa* (mean ± SE). Asterisks at the top of bars indicate significant differences between means of activity enzyme from bees emerged in spring and summer: * *p* < 0.05, ** *p* < 0.001. High quality figures are available online.

The activity of lipase was very low (5.35 nmol fatty acid mg^-1^). Carboxylesterases were much more active, especially C4-esterase. Butyric acid esters (C4) were the best substrate for them. Ester of acetic acid was hydrolyzed at a lower rate. Decomposition of decanoic acid ester was the weakest (Figure 3). A higher activity of the analyzed enzymes of lipid metabolism was seen in the *O. rufa* that emerged in the summer than in those that emerged in spring. For C4 esterase and lipase, the differences were statistically significant.

## Discussion

There are not many data about the metabolism of bees of *Osmia* genus during its ontogenesis. Only decreases of the body weight and reductions of fat body size during overwintering were well documented (Bosch and Kemp 2003; Kemp et al. 2004; Bosch et al. 2010; Sgolastra et al. 2010). Until now, there has been no information about main biomolecules' metabolism and the activity of responsible enzymes. The biochemical consequences of prolonged postdiapause, a procedure commonly used in the commercial rearing of *O. rufa,* were not studied. As *O. rufa* do not intake any food from the environment during the overwintering period, they must rely on energy reserves obtained during larval stage development. Larvae of *O. rufa* feed on pollen with some addition of nectar. As *O. rufa* collect pollen from various types of plants (Wilkaniec et al. 1997), pollen for their larvae may be chemically distinct from each other (Pacinni 1996). This hypothesis is supported by the results of Konrad et al. (2009), who found large variations in sugar content in crops of newly emerged *O. rufa.* The differences in the amount and quality of pollen eaten during larval development can explain the wide range of values of the analyzed parameters, namely body weight and lipid, protein, and total sugar content, which were noted among the individuals in both groups of bees. This suggestion is confirmed by the results of previous studies concerning *O. rufa* body weight and its changes upon different amounts of eaten pollen (Giejdasz 2002; Wilkaniec et al. 2004).

The main reserve materials in insects during dormancy are lipids (Hahn and Delinger 2007). Lipids were also the main energetic material during overwintering *O. rufa.* Lipids constitute 20% the wet weight of the *O. rufa.* Our results coincidence with Buckner et al. (2004), who determined as much as 20% of the body weight to be lipids in diapausing prepupae of *Megachile rotundata,* which belongs to the same family as *O. rufa.* This percentage is considerably higher than has been recorded in other insects studied so far (Fast 1970). Lipids in fat bodis originate from larval diet, and they are also partly synthesized by conversion from carbohydrates (Beenakkers et al. 1985; Canavoso et al 2001; Ziegler and Ibrahim 2001; Hahn and Delinger 2007). The extension of postdiapause lead to a substantial increase of lipase activity and the reduction of lipid content in the body of *O. rufa.* This observation confirmed previous studies (Dmochowska et al. 2011). Lipids stored in the insect's fat bodies are probably used also for the production of reserve materials for oocytes in the ovary of *O. rufa.* An increase in the size and number of oocytes in *O. rufa* takes place during the entire overwintering period (Wasielewski et al. 2011). Intensive lipid mobilization is stimulated by adipokinetic hormone and octopamine by activation in fat body triacylglycerol lipases (Canavoso et al. 2001). Judging by the level of lipase activity in our study, this phenomenon does not occur at moment of emergence of *O. rufa.* In newly emerged *O. rufa,* the activity of lipase was very low and was coincident with high levels of lipids. Both facts may be important for protecting the energy store for the maiden flight of females.

In insects, esterases are involved in important physiological processes, including the catabolism of juvenile hormone (Zera et al. 1992), pesticide resistance (Whyard et al. 1995; Rosario-Cruz et al. 1997), digestion (Kerlin and Hughes 1992; Argentine and James 1995), and reproduction (Richmond and Senior 1991; Karotam and Oakeshott 1993). Carboxylesterases participate in the metabolism of lipid compounds. These enzymes can hydrolyze endogenous substances or promote xenobiotic detoxification (Shen and Dowd 1991). They play an important role in immunity against insecticides and plants' secondary metabolites (Cai et al. 2009). So, the activity of carboxy-lesterases is important for bee health after emergence.

Regardless of the time of emergence, esterases of *O. rufa* showed the highest activity towards esters of butyric acid (C4), and the activity of this esterase increased significantly in summer. High activities of C4, C2, and C10 carboxyesterases were also observed in an APIZym test (Dmochowska et al. 2011). This result is in agreement with that obtained for another solitary bee, *M. rotundata.* Similar to *O. rufa,* esters of aliphatic acids of 3C and 4C length were metabolized by this bee more easily than esters of acids of shorter or longer chains (Frohlich 1990). It was different in *Apis melifera,* whose esterases were more active towards acetic acid esters (C2), and their activity decreased to esters formed by acids with longer aliphatic chains (Dziuban et al. 2010).

In our study, a significantly higher activity of lipase and only slightly higher C2 esterase activity was discovered after prolonged wintering. In *Hyalomma dromedilrii,* these enzymes play a principal role in the interconversion of lipovitellins during embryogenesis (Fahmy et al. 2004). They may play a similar role in *O. rufa* oogenesis.

Glycogen is the main storage carbohydrate in the animal kingdom. In insects, it is synthesized and stored mainly in the fat bodies and muscles. Hypertrehalosemic hormone (HrTH) is responsible for the mobilization of glycogen to glucose, which is essential for further trehalose synthesis, the main sugar of insect hemolymph (Arrese and Soulages 2010). The level of glycogen in emerged *O. rufa* was twice that of hibernating *Osmia cornifrons* (Hoshikawa et al. 1992) and 2 to 3 times lower than that of honey bee workers (Farjan 2008). The low glycogen level in *O. rufa* maybe connected to its transformation into glycerol or trehalose. Both are necessary cryoprotectants to survive freezing weather. This process was observed in *Phyto americanus* and *P. deplanatus* (Ring and Tesar 1980; Ring 1982). Glycogen and total carbohydrate level were lower in bees that emerged in summer.

Total sugar content in emerged *O. rufa* was high and close to the value found in newly emerged honey bee workers (Farjan 2008). The lack of clear differences in total sugar content between *O. rufa* and honey bees is puzzling, as the diet of *A. mellifera* is high in carbohydrates while the diet of *O. rufa* is rich in proteins and lipids. Pollen, the main component of the diet of *O. rufa* larvae, contains mainly proteins and lipids, carbohydrates as starch, and soluble sugars, which constitute only a minor part of its composition (Pacinni 1996; Speranza et al. 1997).

The level of glucose in *O. rufa* was low, which is characteristic for many insects. The fluctuation of glucose in hemolymph is an important signal regulating the rate of metabolism (Arrese and Soulages 2010). The content of glucose was significantly higher in *O. rufa* that emerged in summer. This result was due to a significantly higher activity of trehalase and maltase, which degrade disaccharides to glucose. Krunic and Stanisavljevic (2006) found that during postdiapause, concentrations of cryoprotectants decline significantly, even under constant external temperature condition. Carbohydrates such as trehalose have a dual role as cryoprotectants and sources of energy. Glucose released by the action of trehalase can be built into glycogen or immediately catabolized (Hanh and Delinger 2007). Just after emergence, the activity of *O. rufa* trehalase was clearly lower than maltase and sucrase (Figure 2). Similar findings were observed in newly-emerged honey bee and hawk moth development, and may be an adaptation to a diet appriopriate for an adult individual (Sobiech et al. 1984; Żółtowska et al. 2012). On other hand, low activity of α-amylase was a bit surprising because this enzyme is important in digesting starch from pollen (Ohashi et al. 1999), the main component of *O. rufa* diet. Starvation during the overwintering period may be a reason for low activity of amylase and cellobiase before emergance. Most likely, higher activities of amylase appear only when *O. rufa* eat their first nourishment after emergence, because it is one of the digestive enzymes induced by diet.

Diapause is a dynamic process (Denlinger 2002), and the prolongation of the overwintering period will lead to a higher depletion of energy reservoirs. The results of our study confirmed this hypothesis. As was expected, total carbohydrates, glycogen, fat, and protein content were significantly lower in bees that emerged in July compared to those that emerged in April (Figure 1). The obtained results are in agreement with the earlier studies on other bee species from *Megachilidae* family (Bosch and Kemp 2003, 2004; Sgolastra et al. 2010). The changes in the studied biochemical indicators in *O. rufa* that emerged in summer may have resulted from the acceleration of *O. rufa* metabolism. Such a phenomenon was observed by Kemp et al. (2004), who analyzed oxygen usage in *Osmia lignaria* wintering at a stable temperature of 4° C.

*O. rufa* are worth more detailed biochemical studies because of their usefulness in pollination of crops. Particularly interesting is their life cycle, especially in regards to the possibility of regulating overwintering time. The role of wild bees as alternative pollinators will be more and more important in agriculture due to the decrease of populations of honey bees.

## Abbreviations

**C2**,: 4-Nitrophenyl-acetate;
**C4**,: 4-Nitrophenyl-butyrate;
**C10**,: 4-Nitrophenyl-decanoate
